# Assessing the added value of group B *Streptococcus* maternal immunisation in preventing maternal infection and fetal harm: population surveillance study

**DOI:** 10.1111/1471-0528.16852

**Published:** 2021-08-17

**Authors:** T Lamagni, C Wloch, K Broughton, SM Collin, V Chalker, J Coelho, SN Ladhani, CS Brown, N Shetty, AP Johnson

**Affiliations:** ^1^ Healthcare‐Associated Infection & Antimicrobial Resistance Division National Infection Service Public Health England London UK; ^2^ Respiratory and Vaccine Preventable Reference Unit Bacteriology Reference Department National Infection Service Public Health England London UK; ^3^ Immunisation and Countermeasures Division National Infection Service Public Health England London UK

**Keywords:** England, ethnic groups, population surveillance, pregnancy complications, infectious, *Streptococcus agalactiae*, surgical wound infection

## Abstract

**Objective:**

To assess the incidence of maternal group B *Streptococcus* (GBS) infection in England.

**Design:**

Population surveillance augmented through data linkage.

**Setting:**

England.

**Population:**

All pregnant women accessing the National Health Service (NHS) in England.

**Methods:**

Invasive GBS (iGBS) infections during pregnancy or within 6 weeks of childbirth were identified by linking Public Health England (PHE) national microbiology surveillance data for 2014 to NHS hospital admission records. Capsular serotypes of GBS were determined by reference laboratory typing of clinical isolates from women aged 15–44 years. Post‐caesarean section surgical site infection (SSI) caused by GBS was identified in 21 hospitals participating in PHE SSI surveillance (2009–2015).

**Main outcome measures:**

iGBS rate per 1000 maternities; risk of GBS SSI per 1000 caesarean sections.

**Results:**

Of 1601 patients diagnosed with iGBS infections in England in 2014, 185 (12%) were identified as maternal infections, a rate of 0.29 (95% CI 0.25–0.33) per 1000 maternities and representing 83% of all iGBS cases in women aged 18–44 years. Seven (3.8%) were associated with miscarriage. Fetal outcome identified excess rates of stillbirth (3.4 versus 0.5%) and extreme prematurity (<28 weeks of gestation, 3.7 versus 0.5%) compared with national averages (*P* < 0.001). Caesarean section surveillance in 27 860 women (21 hospitals) identified 47 cases of GBS SSI, with an estimated 4.24 (3.51–5.07) per 1000 caesarean sections, a median time‐to‐onset of 10 days (IQR 7–13 days) and ten infections that required readmission. Capsular serotype analysis identified a diverse array of strains with serotype III as the most common (43%).

**Conclusions:**

Our assessment of maternal GBS infection in England indicates the potential additional benefit of GBS vaccination in preventing adverse maternal and fetal outcomes.

## Introduction

Despite longstanding recognition as a complication of pregnancy and childbirth, few studies have quantified the burden of maternal invasive infection by group B *Streptococcus* (GBS).^1^

Pioneering research by Dr Rebecca Lancefield in the 1930s identified and classified the antigenic properties of beta haemolytic streptococci, opening important new investigative channels.^2^, ^3^ A case series from Queen Charlotte’s in 1938 applied these microbiological techniques to identify GBS as a cause of (fatal) puerperal sepsis.^4^ With the emerging recognition of GBS neonatal infection during the 1970s, the focus switched to the study of infant disease; arguably, GBS as a cause of maternal sepsis became overlooked. As such, our understanding of the epidemiology of GBS maternal sepsis remains limited, despite the overall increases in the incidence of adult disease.^5^

With the global drive to develop a GBS vaccine, studies are underway in many countries to characterise GBS epidemiology ahead of licensure. Although the prevention of infant GBS disease is the main target, maternal immunisation will also bring potential direct benefits to the mother.^6^ A recent UK study estimated the incidence of severe maternal sepsis from GBS at 0.037 per 1000 maternities,^7^ although a more comprehensive enumeration of the burden of maternal infection has not been undertaken. Quantifying this will provide a more comprehensive estimate of the potential cost‐effectiveness of maternal immunisation given that immunisation is likely to provide protection for both neonate and mother.

In their Group B Streptococcus Vaccine Development Technology Roadmap, the World Health Organization highlight the need for further research to assess the burden on maternal disease in preparation for the advent of an effective licensed vaccine.^6^ Our study aimed to quantify the incidence of GBS as a cause of invasive infection and surgical site infection after caesarean section in England.

## Methods

The data used for the study were drawn from Public Health England (PHE) surveillance and National Health Service (NHS) Digital Hospital Episode Statistics.

### Case definitions

Maternal invasive GBS (iGBS) infection was defined by the isolation of GBS from a normally sterile site in women identified as being pregnant or within 6 weeks of delivery (live or stillbirth) at the time of diagnosis.

Caesarean section GBS surgical site infection (SSI) was defined based on clinical and laboratory findings and diagnosed within 30 days of surgery.^8^ Infections were classified as superficial incisional, deep incisional and organ/space (endometritis or other reproductive tract infections).^9^

### Data sources

National laboratory surveillance records captured through PHE’s Second Generation Surveillance System (SGSS) were used to identify patients in England diagnosed with iGBS infection in 2014.^10^ The results of serotyping GBS isolates submitted to the PHE national reference microbiology laboratory were used to characterise isolates from women of childbearing age (15–44 years). National guidelines request the reporting and submission of GBS isolates from normally sterile sites, although both are undertaken on a voluntary basis. Laboratory surveillance is subject to weekly audits of participation and data quality.^11^

Records of NHS hospital admissions from 2013 to 2015 in England (NHS Digital Hospital Episode Statistics, HES^©^) were used to identify maternity admissions occurring prior to and after the iGBS episodes identified in the SGSS data. HES captures NHS‐funded maternity care in England, including home births.

The PHE caesarean section SSI surveillance data were captured by NHS hospitals participating in voluntary surveillance.^8^ Women undergoing caesarean section between 2009 and 2015 were included. Caesarean section SSIs were captured using the standard surveillance methodology used in England, which is based on the definitions from the Centers for Disease Control and Prevention (CDC).^8^ Women were prospectively followed‐up during their inpatient stay by trained hospital surveillance staff to detect SSIs. Infections arising post‐discharge were detected through readmission to hospital, community midwife reporting or via a patient questionnaire at 30 days after the operation.

### Data analysis

Laboratory‐confirmed iGBS infection records were extracted from SGSS and submitted to the NHS Demographics Batch Service to complete/check the unique patient identifiers (NHS number) and were then linked to HES using the NHS numbers. All linked hospital admission records were analysed to identify pregnancy or childbirth through the completion of any of the following fields: (i) maternity data fields (BIRSTAT, DOBBABY, DELMETH); (ii) clinical coding fields (International Classification of Diseases, tenth revision (ICD‐10), chapter XV, O00–O99, ‘Pregnancy, childbirth and the puerperium’); (iii) admission method (maternity); and (iv) operative procedure codes (OPCs R14, R15, R17, R18, R19, R2*). Laboratory‐confirmed iGBS cases up to 280 days prior to or 42 days after delivery were considered maternal cases. Details of hospital admissions closest in time to iGBS infection diagnosis were analysed to assess length of stay. Outcomes in GBS cases were compared with normative birth statistics for stillbirth, preterm delivery and extreme prematurity for 2014 (*n* = 664 149; gestation recorded for 660 944 births) from the Office for National Statistics (ONS) and for delivery method from NHS Digital maternity data for 2014/15 (*n* = 636 643; delivery method recorded for 627 576 women). Rates of iGBS disease per 1000 maternities were calculated using ONS maternity denominators for England for 2014 (*n* = 638 863). Rates by ethnic group used NHS Digital deliveries in NHS hospitals in 2014/15 as the denominator, as ONS data for all maternities in England were not available broken down by ethnic group.

The risk of GBS SSI in women undergoing caesarean section was estimated by multiplying the observed proportion of SSIs with a microbiological diagnosis of GBS infection by the total number of SSIs, with the latter including clinically defined infections meeting the surveillance case definitions.

Isolates submitted to the national reference laboratory were characterised according to their capsular polysaccharide serotype using a modified version of the Statens Serum Institut (Copenhagen, Denmark) GBS latex test.^12^

### Statistical analysis

Confidence intervals (95% CIs) and χ^2^ tests were calculated using stata 13.1 (Stata Corporation, College Station, TX, USA), with binomial and Poisson distributions used for 95% CIs around proportions and rates, respectively.

There was no patient involvement in the development of the study.

## Results

### Maternal invasive GBS infection

Laboratory surveillance identified 1601 patients diagnosed with iGBS infection in England in 2014. Of these, 1563 (98%) had records containing an NHS number and 1546 (97%) were successfully linked to one or more hospital admission records. Analysis of clinical and admission information identified 185 (12%) as infections arising during pregnancy, around the time of delivery or within 6 weeks postpartum. This equated to an incidence of maternal iGBS infection of 0.29 per 1000 maternities (Table S1). All women had GBS‐positive blood cultures (Table S2). Amongst all iGBS cases in women aged 18–44 years, 83% were maternal cases; these 185 cases accounted for most of the excess number of cases in women versus men in this age group (222 versus 47; Table S1). Compared with non‐maternity cases, pregnant women had 78 times the rate of iGBS infection (RR 78.21, 95% CI 54.7–114.5).

The median age of maternal GBS cases was 30 years (range 18–44 years), with an age distribution that was similar to that for all maternities in England (Figure S1). Recorded ethnicity (*n* = 174; Table S3) identified a higher rate of infections in Asian (0.61/1000, RR 2.76, *P* < 0.001), Chinese/Other (0.60, RR 2.69, *P* < 0.001) and black (0.55, RR 2.50, *P* < 0.001) women, compared with white women (0.22) or women of mixed ethnicity (0.20).

Gestational age at the time of GBS diagnosis was recorded for 134 women, with 96% (129) of iGBS infections diagnosed in the third trimester, one in the first trimester (1%) and four in the second trimester (3%) (Table 1). Just one of the women was recorded as having a multiple birth (twins). Details of delivery (*n* = 144) identified 42% (61) of cases as having undergone an emergency caesarean section (cf. 15% in England, *P* < 0.001); 79 (55%) were vaginal deliveries and one an elective caesarean section (Table 1). Most maternal GBS infections were diagnosed on the day (74%, 124/168) or within 1 day of delivery (93%); ten (6%) were diagnosed ≥2 days postpartum (Figure S2; Table 1).

**Table 1:** Obstetric features of maternal invasive GBS infection, England 2014

	Invasive GBS infection (*n* = 185)			All births & maternities in England*	*P* (χ^2^)
	No.	(%)	(95% CI)	(%)	
Trimester at diagnosis (*n* = 134)					
First (0–12 weeks of gestation)	1	(0.7%)	(0.0–4.1%)		
Second (13–27 weeks of gestation)	4	(3.0%)	(0.8–7.5%)		
Third (28–40 weeks of gestation)	129	(96.3%)	(91.5–98.8%)		
Diagnosis to delivery interval, days (*n* = 168)					
Antepartum (−4 to −2 days)	1	(0.6%)	(0.0–3.3%)		
Peripartum (−1 to 1 days)	157	(93.5%)	(88.6–96.7%)		
Postpartum (2 to 26 days)	10	(6.0%)	(2.9–10.7%)		
Delivery method (*n* = 144)					
Vaginal	79	(54.9%)	(46.4–63.2%)	(73.5%)	
Elective caesarean section	1	(0.7%)	(0.0–3.8%)	(11.1%)	
Emergency caesarean section	61	(42.4%)	(34.2–50.9%)	(15.4%)	<0.001
Pregnancy outcome (*n* = 184)					
Miscarriage	7	(3.8%)	(1.5–7.7%)		
Live or stillborn	174	(94.6%)	(90.2–97.4%)		
Fetal outcome (*n* = 147)					
Stillborn	5	(3.4%)	(1.1–7.8%)	(0.5%)	<0.001
Liveborn	142	(96.6%)	(92.2–98.9%)		
Completed gestation (*n* = 134)					
Preterm liveborn (<37 weeks)	10	(7.5%)	(3.6–13.3%)	(7.8%)	
Extreme preterm liveborn (<28 weeks)	5	(3.7%)	(1.2–8.5%)	(0.5%)	<0.001
Diagnosis in relation to maternity admission (*n* = 185)					
Diagnosis in childbirth admission	171	(92.4%)			
Median duration of stay: days, range, (IQR)	7	1–20 (5–9)			
Diagnosis in non‐childbirth admission	13	(7.0%)			
Median admission stay: days, range, (IQR)	4	2–7 (3–5)			

*Data for gestational age and fetal outcome in England sourced from Office for National Statistics birth statistics for 2014; delivery method sourced from 2014/15 NHS Digital maternity data.

The GBS diagnosis was made during admission for childbirth in 92% (171) of cases. The median length of stay for these women was 7 days (IQR 5–9 days) (Table 1). For 13 women (7%), the admission closest to the time of GBS diagnosis was separate from the admission when childbirth occurred (eight antepartum, five postpartum), with a median length of stay of 4 days (IQR 3–5 days).

**Figure 1:**
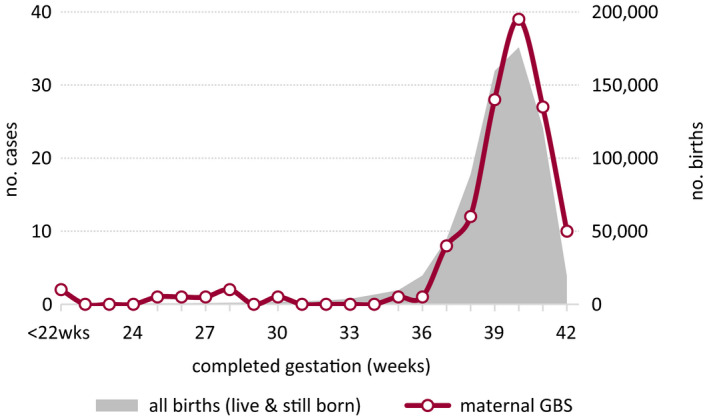
Distribution of maternal invasive GBS cases according to gestational age of infant versus all births*, England 2014. *Sourced from the Office for National Statistics.

Analysis of clinical coding identified that pregnancies for seven women (3.8%) with iGBS infection concluded with miscarriage. Of the remaining 174 cases, fetal outcome was reported for 147 women (84%), with stillbirth recorded in five cases (3.4%; two antepartum, one intrapartum, two indeterminate), significantly higher than the national average of 0.5% (*P* < 0.001). Completed gestation was reported for 134 women, ten of whom recorded preterm delivery (<37 weeks of gestation; Figure 1), similar to the national rate (7.5 versus 7.8%). However, five of these recorded extreme prematurity (<28 weeks of gestation), which at 3.7% was significantly higher than the 0.5% rate nationally (*P* < 0.001).

No maternal deaths associated with GBS infection were identified.

### Microbiological characteristics of strains

**Figure 2:**
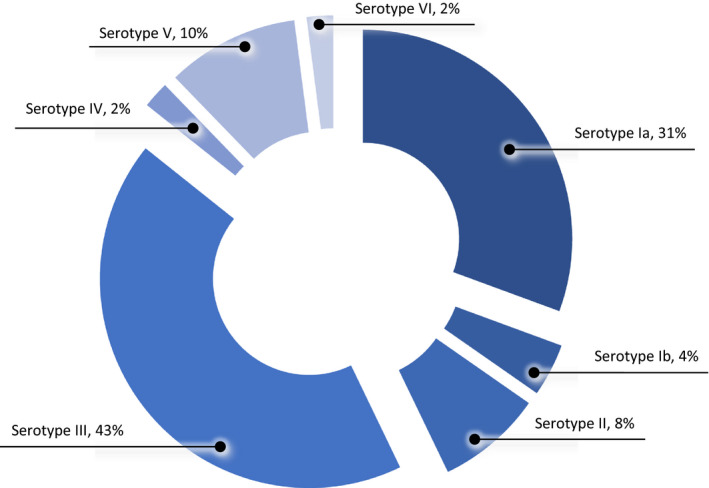
Distribution of GBS capsular serotypes in women aged 15–44 years, England 2014.

Analysis of clinical GBS isolates submitted to the national reference laboratory in 2014 identified 49 referrals from women aged 15–44 years. Forty‐five were recorded from normally sterile sites, three were from placenta and one was from an unknown site. Capsular serotype analysis identified a diverse array of strains (Figure 2), with serotype III being the most common (*n* = 21, 43%) followed by serotype Ia (*n* = 15, 31%).

### Caesarean section surgical site infection

Data for 27 860 women undergoing caesarean section were submitted by 21 hospitals in England between 2009 and 2015. Of these procedures, 7.8% (2180) resulted in SSI within 30 days of surgery. GBS accounted for 5.4% (47/868) of all microbiologically confirmed SSIs, translating to an estimated GBS risk of 4.2 (95% CI 3.5–5.1) per 1000 operations (Table S4).

**Figure 3:**
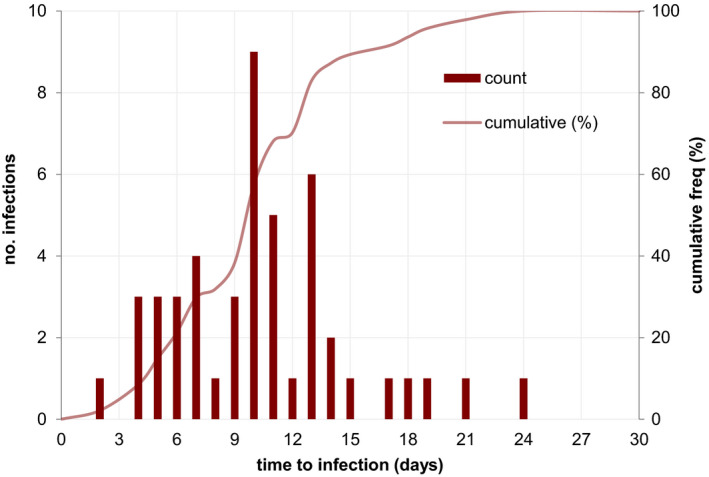
Distribution of interval between caesarean section and GBS surgical site infection, 2009–2015.

The median time‐to‐onset of GBS SSI was 10 days (IQR 7–13 days) post‐caesarean section (Figure 3). Most GBS infections (35, 74%) were classed as superficial incisional infections, with two (4%) identified as deep incisional and ten (21%) identified as organ‐space infections. One in five (10, 21%) of these infections required hospital readmission for management of the infection.

There were no observable differences in GBS infection risk according to the caesarean section urgency of risk category (*P* = 0.41; Table S5). The risk of GBS infection increased significantly with increasing BMI (Figure S3), reaching 49 per 1000 in women whose BMI was ≥35 kg/m^2^.

## Discussion

### Main findings

Our study is the first comprehensive assessment of the incidence of maternal GBS infection. Through the linkage of laboratory surveillance data to hospital admission records, we identified a significant burden of maternal GBS sepsis, affecting 1 in 3000 women (0.29/1000), which is 78 times higher than the background rate in women of childbearing age. This builds on Kalin et al.’s assessment of GBS sepsis in 2011/12, restricted to cases of clinically severe sepsis, which reported an incidence of 0.037 per 1000 women.^7^ Although comparison with other countries is hampered by a dearth of studies, we note that our estimate is similar to a contemporary study in France (0.32 per 1000 maternities) but is higher than that seen in the USA (0.10 per 1000 maternities).^1^, ^13^, ^14^

We identified pregnancy outcomes to be significantly worse than background population rates, with excess rates of emergency caesarean section, stillbirth and extreme prematurity, highlighting the additional personal and economic impact of these infections. Although we cannot definitively attribute GBS infection as the cause of these outcomes, ascending GBS has been identified as a cause of uterine inflammation, inducing labour and leading to spontaneous abortion and preterm birth. Even with the limited data available, an estimated 1% of stillbirths in developed countries and 4% in sub‐Saharan Africa are thought to be caused by GBS.^15^ Our understanding of the aetiological fraction of these devastating outcomes apportionable to GBS will remain unknown until a maternal immunisation programme is introduced and its impact on the occurrence of adverse outcomes such as stillbirth measured.

We noted disparities in the rates of iGBS infection according to ethnicity, with women of Asian, Black or Chinese/Other ethnicity experiencing rates that were twice as high as those in women of white or mixed ethnicity. Global research into GBS suggests higher rates of rectovaginal GBS carriage in African countries than in Europe, and still lower rates in women from Southern and Eastern Asia.^16^, ^17^ A similar pattern was identified in a recent antenatal screening study from London.^18^ Our study findings are not entirely consistent with this, however, as rates of disease in Asian women were higher than for white counterparts. The differences that we observed are of concern and warrant further investigation.

Although typically not severe, we identified a sizeable role for GBS as a cause of post‐caesarean section SSI, affecting 4 per 1000 women. A recent systematic review estimated 9.7% of organ/space SSIs post‐caesarean section to be caused by GBS.^19^ Our study similarly found a relatively higher proportion of organ/space infections compared with other pathogens.^8^ Our findings highlight the excess risk of GBS SSI in women who are overweight, rising to 49 per 1000 women with a BMI greater than 35 kg/m^2^. Identifying opportunities for the prevention of caesarean section wound infections should be a priority given that one in five GBS infections resulted in hospital readmission.

### Strengths and limitations

Our study provides a comprehensive estimate of the burden of maternal GBS infection. Through the access and linkage of national laboratory surveillance data to hospital admission records, we have been able to estimate the incidence of maternal iGBS disease across England. In a modest number of study sites, we were further able to assess the incidence and characteristics of GBS surgical site infection. In combination, these provide a more robust assessment of the importance of GBS as a cause of maternal infection to date.

We did not assess the direct or indirect costs associated with GBS sepsis or wound infection but note the median length of a stay for women diagnosed with invasive GBS infection during their maternity admission being 7 days, the additional stays in hospital for women whose sepsis onset was earlier in their pregnancy, or occurred post discharge, and the readmissions for management of postoperative wound infections. The adoption of routine surveillance for caesarean section infection should be considered by hospitals, with a recent economic analysis highlighting the considerable savings achievable through reductions in wound infection rates.^20^

The reliance on microbiological detection of GBS infection and accuracy of clinical coding undoubtedly results in an under‐detection of cases overall, and therefore incidence estimates in this study are likely to underestimate maternal GBS infection.^21^ Our assessment of isolates submitted to the national reference laboratory provides additional information of value in considering the potential breadth of coverage for current and future vaccine candidates. Although we do not have information to confirm that these isolates were associated with maternal infection, our study identified 83% of women aged 18–44 years with invasive GBS infection to be pregnant or postpartum. All laboratories in England are required to submit all GBS sterile site isolates to the national reference laboratory; however, as illustrated in our analysis, only a small proportion of isolates were submitted in practice. Although this introduces the potential for selection bias, which could influence the serotype distribution, it is also likely to reflect individual hospital practices, whereby only some hospital laboratories routinely submit their isolates for serotyping.

### Interpretation

Maternal vaccination with the aim of preventing infant GBS disease offers a potentially valuable opportunity for additionally preventing invasive maternal GBS infections, as most occur beyond 28 weeks of gestation. Following antenatal vaccination, immunological protection up to 1 month postpartum would potentially prevent SSIs post‐caesarean section as well as maternal sepsis cases. Our assessment of a small number of capsular serotypes in women of childbearing age identified a diverse range of serotypes, potentially more diverse than those associated with early‐onset disease.^5^ Nonetheless, 98% of isolates belonged to serotypes included in the hexavalent GBS vaccine currently under trial (serotypes Ia, Ib, II, III, IV and V; ClinicalTrials.gov NCT03170609).^22^ Including maternal outcomes in vaccine cost‐effectiveness assessments is of critical importance given the not insubstantial economic burden likely to be associated with such infections.

## Conclusion

Our assessment captured valuable data on the burden of maternal GBS infection in England, indicating the potential of antenatal GBS vaccination to prevent adverse maternal and fetal outcomes associated with this pathogen. Increasing referral of maternal isolates to the national reference laboratory would further assist in our understanding of the epidemiology of these infections and provide an important step in preparing for the advent of maternal GBS vaccination.

### Disclosure of interests

None declared. Completed disclosure of interests form available to view online as supporting information.

### Contribution to authorship

TL was principal investigator and led the writing of this article. All authors assisted with the design of the study. TL undertook a background literature review and drafted the article. All authors contributed to the interpretation of the results and the critical review of the article.

### Details of ethical approval

Public Health England has NHS Health Research Authority (HRA) Confidentiality Advisory Group (CAG) approval for the collation of surveillance data, in accordance with section 251 of the NHS Act 2006. No additional ethical approval was required to undertake this study.

### Funding

This study was funded by PHE, which received no external funding for the analysis. The corresponding author had full access to all the data in the study and had final responsibility for the decision to submit for publication.

### Acknowledgements

We extend our thanks to all the microbiologists who submitted strains to the national reference laboratory and PHE colleagues for their assistance with serotyping. In addition, we acknowledge Anne‐Marie O’Connell’s expert management of the Second Generation Surveillance Service and data management support provided by Nick Hinton. Hospital Episode Statistics© 2016 were reused with the permission of NHS Digital.

## Supporting information


**Table S1.** Number and rate of invasive GBS infection diagnoses by sex and maternity, England 2014.
**Table S2.** Specimen source of *Streptococcus agalactiae* isolate in women with maternal invasive infection, England 2014.
**Table S3.** Maternal invasive GBS infection according to ethnicity, England 2014.
**Table S4.** GBS surgical site infection (SSI) risk in women undergoing caesarean section, England 2009–2015.
**Table S5.** GBS surgical site infection (SSI) risk according to urgency of caesarean section, England 2009–2015.
**Figure S1.** Age distribution of maternal GBS cases versus all maternities, England 2014.
**Figure S2.** Distribution of maternal GBS cases according to timing of onset in relation to delivery, England, 2014.
**Figure S3.** BMI‐stratified GBS SSI risk in women undergoing caesarean section, 2009–2015.

Supplementary Material

Supplementary Material

Supplementary Material

Supplementary Material

Supplementary Material

Supplementary Material

Supplementary Material

Supplementary Material

Supplementary Material

Supplementary Material

## Data Availability

Data available on request from the authors.
