# Supplementary material for: Assessing the added value of group B Streptococcus maternal immunisation in preventing maternal infection and fetal harm: population surveillance study
Source: BJOG. 2021 Aug 17;129(2):233–40. doi: 10.1111/1471-0528.16852 (PMC9291181; doi:10.1111/1471-0528.16852)
Supplement: Supplementary file 1 — Table S1. Number and rate of invasive GBS infection diagnoses by sex and maternity, England 2014. Table S2. Specimen source of Streptococcus agalactiae isolate in women with maternal invasive infection, England 2014. Table S3. Maternal invasive GBS infection according to ethnicity, England 2014. Table S4. GBS surgical site infection (SSI) risk in women undergoing caesarean section, England 2009–2015. Table S5. GBS surgical site infection (SSI) risk according to urgency of caesarean section, England 2009–2015. Figure S1. Age distribution of maternal GBS cases versus all maternities, England 2014. Figure S2. Distribution of maternal GBS cases according to timing of onset in relation to delivery, England, 2014. Figure S3. BMI‐stratified GBS SSI risk in women undergoing caesarean section, 2009–2015. [file BJO-129-233-s006.docx]

SUPPORTING INFORMATION

**Table S1** Number and rate of invasive GBS infection diagnoses by sex and maternity, England 2014

|  | **No.** | **(%)** |  | **(95% CI)** | | |
| --- | --- | --- | --- | --- | --- | --- |
| Total no. cases | 1601 |  |  |  |  |  |
| Mid-year population estimate | 54,316,618 |  |  |  |  |  |
| Rate per 1000 population | 0.029 |  |  | (0.03 | - | 0.03) |
|  |  |  |  |  |  |  |
| No. cases in women 15-44y | 222 | (13.9%) |  |  |  |  |
| Mid-year population estimate women 15-44y | 10,631,532 |  |  |  |  |  |
| Rate per 1000 women 15-44y | 0.021 |  |  | (0.02 | - | 0.02) |
|  |  |  |  |  |  |  |
| No. cases with hospital record | 1546 | (96.6%) |  |  |  |  |
| No. maternal cases | 185 | (12.0%) |  |  |  |  |
| No. maternities | 638,863 |  |  |  |  |  |
| Rate per 1000 maternities | 0.290 |  |  | (0.25 | - | 0.33) |
|  |  |  |  |  |  |  |
| No. non-maternal cases 15-44y | 37 |  |  | (0.00 | - | 0.01) |
| Rate non-maternal women 15-44y | 0.004 |  |  | (0.00 | - | 0.01) |
|  |  |  |  |  |  |  |
| Rate ratio maternal vs non-maternal | 78.21 |  |  | (54.71 | - | 114.54) |
|  |  |  |  |  |  |  |
| No. cases in men 15-44y | 47 | (2.9%) |  |  |  |  |
| Mid-year population estimate men 15-44y | 10,734,838 |  |  |  |  |  |
| Rate per 1000 men 15-44y | 0.004 |  |  | (0.00 | - | 0.01) |
|  |  |  |  |  |  |  |
| Rate ratio men vs women (non-maternal) 15-44y | 1.18 |  |  | (0.75 | - | 1.87) |
|  |  |  |  |  |  |  |

**Table S2** Specimen source^*^ of *Streptococcus agalactiae* isolate in women with maternal invasive infection, England 2014

| N=185 | no. | (%) |
| --- | --- | --- |
| Blood | 185 | (100.0%) |
| Lower genital tract | 5 | (2.7%) |
| Urine | 4 | (2.2%) |
| Placenta | 3 | (1.6%) |
| Swab^**^ | 3 | (1.6%) |
| Upper genital tract | 1 | (0.5%) |

**Streptococcus agalactiae* may have been isolated from more than one source in each patient.

** not further specified

**Table S3** Maternal invasive GBS infection according to ethnicity, England 2014

| Ethnicity (n=174) | No. cases | (%) | rate per 1000 deliveries* | (95% CI) | rate ratio | (95% CI) |
| --- | --- | --- | --- | --- | --- | --- |
| British (White) | 80 | (43.2%) |  |  |  |  |
| Any other White background | 20 | (10.8%) |  |  |  |  |
| White | **100** | **(54.1%)** | **0.22** | (0.18-0.27) | 1.00 |  |
|  |  |  |  |  |  |  |
| Bangladeshi (Asian or Asian British) | 7 | (3.8%) |  |  |  |  |
| Any other Asian background | 5 | (2.7%) |  |  |  |  |
| Indian (Asian or Asian British) | 13 | (7.0%) |  |  |  |  |
| Pakistani (Asian or Asian British) | 16 | (8.6%) |  |  |  |  |
| Asian | **41** | **(22.2%)** | **0.61** | (0.44-0.83) | 2.76 | (1.92-3.97) |
|  |  |  |  |  |  |  |
| Any other Black background | 4 | (2.2%) |  |  |  |  |
| African (Black or Black British) | 10 | (5.4%) |  |  |  |  |
| Caribbean (Black or Black British) | 2 | (1.1%) |  |  |  |  |
| Black | **16** | **(8.6%)** | **0.55** | (0.32-0.90) | 2.50 | (1.48-4.24) |
|  |  |  |  |  |  |  |
| Any other Mixed background | 1 | (0.5%) |  |  |  |  |
| Any other ethnic group | 9 | (4.9%) |  |  |  |  |
| Chinese (other ethnic group) | 5 | (2.7%) |  |  |  |  |
| Chinese & other | **15** | **(8.1%)** | **0.60** | (0.33-0.98) | 2.69 | (1.56-4.63) |
|  |  |  |  |  |  |  |
| White and Asian (Mixed) | 1 | (0.5%) |  |  |  |  |
| White and Black Caribbean (Mixed) | 1 | (0.5%) |  |  |  |  |
| Mixed | **2** | **(1.1%)** | **0.20** | (0.02-0.72) | 0.90 | (0.22-3.64) |
|  |  |  |  |  |  |  |
| Missing | **9** | **(4.9%)** |  |  |  |  |
| Total | **185** | **(100.0%)** | **0.29** | (0.25-0.34) |  |  |

*rates calculated using NHS Digital deliveries in NHS hospitals (England), 2014/15

**Table S4** GBS surgical site infection (SSI) risk in women undergoing caesarean section, England 2009-15

| N=27,860 |  |  | |  | |
| --- | --- | --- | --- | --- | --- |
|  | **No.** | (%) | (95% CI) | | |
| All caesarean section SSIs | 2,180 | (7.8%) | (7.5% | - | 8.1%) |
| Microbiologically diagnosed | 868 | (39.8%) |  |  |  |
| GBS SSI | 47 | (5.4%) | (4.0% | - | 7.1%) |
| Estimated no. GBS SSI | 118 |  |  |  |  |
| Estimated risk GBS SSI per 1000 women | 4.24 |  | (3.51 | - | 5.07) |
|  |  |  |  |  |  |
| *Clinical characteristics of GBS SSI (n=47)* |  |  |  |  |  |
| Focus of infection |  |  |  |  |  |
| Superficial incisional | 35 | (74.5%) | (59.7% | - | 86.1%) |
| Deep incisional | 2 | (4.3%) | (0.5% | - | 14.5%) |
| Organ/space | 10 | (21.3%) | (10.7% | - | 35.7%) |
|  |  |  |  |  |  |
| Readmission due to infection | 10 | (21.3%) | (10.7% | - | 35.7%) |
|  |  |  |  |  |  |

**Table S5** GBS surgical site infection (SSI) risk according to urgency of caesarean section, England 2009-15

| N=27,544 |  | No. women | No. SSI | SSI risk per 1000 | (95% CI) | | |  |
| --- | --- | --- | --- | --- | --- | --- | --- | --- |
|  |  |  |  |  |  |  |  | |
| *Maternal or foetal compromise* | Immediate threat to life of woman or foetus | 2,660 | 3 | 1.13 | (0.23 | - | 3.29) | |
|  | Not immediately life-threatening | 10,109 | 20 | 1.98 | (1.21 | - | 3.05) | |
|  |  |  |  |  |  |  |  | |
| *No maternal or foetal compromise* | Requires early delivery | 3,703 | 9 | 2.43 | (1.11 | - | 4.61) | |
|  | At a time to suit the woman and maternity services | 11,072 | 15 | 1.35 | (0.76 | - | 2.23) | |
|  |  |  |  |  |  |  |  | |

Figure S1 **Age distribution of maternal GBS cases vs all maternities*, England 2014**

* sourced from Office for National Statistics (England and Wales)

Figure S2 **Distribution of maternal GBS cases according to timing of onset in relation to delivery, England, 2014**

**Figure S3.** BMI stratified GBS SSI risk in women undergoing caesarean section, 2009-2015
